# Literary Work

**Published:** 1907-09-07

**Authors:** 


### LITERARY WORK.

There is a wide scope for the medical man who has literary aspirations and who desires to contribute to the medical press, or to augment his income by devoting his leisure time to medical literary work. Needless to say, literary work for the medical man is as onerous, as hard, and as disappointing, until an effective start has been made, as it is for the literary layman. So much is daily written that is, from a literary point of view, comparatively worthless, that there appears to be an impression in the minds of most that the style of writing does not matter so long as the subject is of professional interest. No greater mistake can be made than that. An article which is attractively written bears its own commendation, and a little attention paid to points of style and treatment generally brings its own reward. All the medical papers accept articles of professional interest: a few, like The Hospital and some American medical journals, pay ordinary newspaper rates for accepted contributions. Suitable articles are also accepted by the lay press, in which case the remuneration is often far higher than that given by the professional journals. All articles forwarded for consideration should be typewritten or, at least, clearly penned with a wide margin, and it should be a rule of writers never to forward a contribution without a stamped and properly addressed envelope for its return in case of unsuitability.

Both for literary and research work the graduate must have access to good libraries, and in every centre there are excellent collections of books and papers which are available for reference. In London the reading-room of the British Museum is open to practitioners, who may obtain a reader's ticket, usually for three months, on making written application to the Director. The library of the Royal College of Surgeons, Lincoln's Inn Fields, is open to all practitioners who are fellows or members of the College, and to students and others who have a card of introduction from a fellow or member. The libraries of the Royal Medical and Chirurgical Society and of the Royal College of Physicians is available to readers provided with an introduction from a member. The valuable collections in the Bodleian Library at Oxford, in the Royal College of Surgeons, Edinburgh, and at Dublin, are open to graduates on application to the Librarians.

